# Spatially resolved single cell analysis suggests an APOE NCF1 associated immunosuppressive niche and its prognostic signature in thyroid cancer

**DOI:** 10.1007/s12672-026-05061-6

**Published:** 2026-05-30

**Authors:** Han Su, Zheng Zhang, Nuo Li, Zimo Zhang, Renju Zhang, Ziyi Li, Fen Chen, Xinyue Wang, Shuyuan Zhang, Chao Zhang

**Affiliations:** 1 Department of Otolaryngology Head and Neck Surgery, The Second Affiliated Hospital of Anhui Medical University, Hefei, China; 2 First School of Clinical Medicine, Anhui Medical University, No 81 Meishan Road, Hefei, 230032 Anhui China

**Keywords:** Thyroid carcinoma, Single-cell RNA sequencing, Spatial transcriptomics, CD8⁺ T cell exhaustion, *APOE-NCF1* axis, Prognostic model

## Abstract

**Background:**

Thyroid carcinoma (TC) presents a rising global incidence, with a subset of cases progressing aggressively despite standard therapies. The tumor microenvironment (TME), particularly the functional state of CD8⁺ T cells, is crucial in disease progression, yet a systematic, high-resolution understanding of the cellular interactions associated with immune exhaustion and tumor evolution in TC remains limited.

**Methods:**

To address this, we performed an integrated single-cell and spatial transcriptomic analysis of human TC samples. Our approach combined scRNA-seq data processing, cell communication inference, spatial co-localization validation, and machine learning-based prognostic modeling.

**Result:**

We identified a distinct tumor cell subcluster (Trem/T01) characterized by *APOE* expression and a CD8⁺ exhausted T cell (Tex) subset. Computational and spatial analyses revealed a potential *APOE* (from Trem)-*NCF1* (from CD8⁺ Tex) interaction axis, with these cell types demonstrating significant spatial proximity in tumor tissues. Furthermore, we derived a prognostic gene signature from this network, constructing a robust risk stratification model validated across independent cohorts.

**Conclusion:**

This study uniquely delineates a specific tumor-immune interactive niche in TC, providing insights into potential mechanisms into immune evasion via the *APOE-NCF1* axis and delivering a translatable framework for patient prognostication.

**Supplementary Information:**

The online version contains supplementary material available at 10.1007/s12672-026-05061-6.

## Introduction

Thyroid carcinoma (TC), the most prevalent malignancy of the endocrine system, has witnessed a sustained global increase in incidence over the past several decades [1, 2]. While the widespread adoption of high-resolution ultrasonography and other imaging modalities has contributed to elevated detection rates [3], a genuine rise in the disease burden remains undeniable. Clinically, the vast majority of cases are well-differentiated thyroid cancers (DTCs) with favorable prognoses, for which long-term survival is typically achieved through surgical resection, radioiodine (RAI) therapy, and TSH suppression [4]. Nonetheless, significant clinical challenges persist: a subset of DTC patients experience recurrence, dedifferentiation, or distant metastasis [5], while RAI-refractory thyroid cancers and anaplastic thyroid carcinoma (ATC) confront an even grimmer reality marked by limited therapeutic options and exceedingly poor outcomes [6, 7]. Although the current AJCC/TNM staging system and ATA risk stratification have proven instrumental in guiding clinical decision-making [8, 9], their reliance primarily on clinicopathological parameters limits their capacity to fully capture the intricate complexity of tumor biology. Consequently, a deeper interrogation of the fundamental mechanisms driving thyroid cancer progression, coupled with the identification of decisive tumor microenvironmental features that dictate clinical trajectory, is paramount for the discovery of more robust risk stratification biomarkers and novel therapeutic targets.

The tumor microenvironment (TME) is recognized as a pivotal determinant in the progression and therapeutic response of thyroid carcinoma [10]. Therein, the intensity, compositional profile, and functional status of immune cell infiltration are intricately linked to tumor evolution [11]. Among various immune populations, CD8⁺ T cells, serving as central effector units of anti-tumor immunity, directly influence the efficacy of immune surveillance and eradication [12]. However, during tumor progression, sustained antigen exposure and an immunosuppressive milieu frequently drive CD8⁺ T cells into a state of functional decline, a condition termed “exhaustion“ [13]. Exhausted CD8⁺ T cells (CD8⁺ Tex) represent a prototypical dysfunctional state within the TME, orchestrated by persistent antigenic stimulation, chronic inflammatory signaling, and immunosuppressive cues [14]. In contrast to their effector or memory counterparts, CD8⁺ Tex exhibit attenuated cytotoxic potential, restricted proliferative capacity, and a coordinated upregulation of multiple inhibitory receptors (e.g., PD-1, CTLA-4, LAG3, TIGIT, TIM-3), accompanied by distinct transcriptional regulatory networks and metabolic reprogramming [15]. Research indicates that tumors can induce CD8⁺ T cell exhaustion through diverse mechanisms, thereby facilitating immune evasion and disease progression [16]. Given the complex web of cellular communication between CD8⁺ Tex and tumor cells, alongside other immune and stromal components, the mechanisms governing their formation and maintenance likely exert direct influence on immune escape, tumor advancement, and patient prognosis [17]. Consequently, fine-grained phenotyping of these cells and delineating pivotal intercellular axes hold significant mechanistic and translational implications.

Single-cell RNA sequencing (scRNA-seq) enables the characterization of tumor tissue at single-cell resolution, profiling its cellular composition, transcriptional states, and lineage relationships, thereby offering a powerful tool for deciphering tumor cell subpopulations and the functional status of immune cells [18]. Concurrently, spatial transcriptomics (ST) captures transcriptomic profiles while preserving native tissue architecture, thereby revealing the spatial distribution of cell populations and their potential niches within the tissue [19]. Integrative analysis of scRNA-seq and ST data not only facilitates the identification of key cellular subsets but also allows for the validation of their spatial co-localization. This approach yields a more comprehensive evidentiary framework for elucidating the cascade linking cellular identity, intercellular crosstalk, spatial organization, and clinical phenotype.

Nevertheless, conventional bulk transcriptomic sequencing, which averages signals across entire tissue samples, fails to resolve the characteristics of distinct cellular subpopulations or their interactions, and provides no information regarding spatial relationships within the tissue. This fundamentally limits a deeper understanding of the mechanisms underlying immune evasion and tumor progression in thyroid carcinoma. Specifically, CD8⁺ Tex in thyroid cancer remains insufficiently dissected at a systematic level, with a lack of identified, translatable molecular targets.

To address this, the present study investigates the cellular heterogeneity and cell-cell communication networks within the thyroid cancer TME through an integrated single-cell and spatial transcriptomic analysis. We began with systematic processing and annotation of thyroid cancer single-cell data, with a particular focus on elucidating the molecular signatures and functional states of the CD8⁺ Tex subpopulation. Building upon this, we identified a distinct tumor cell subcluster, termed T01. Differential expression analysis revealed that tumor cells within this cluster uniquely express *APOE*. As a key apolipoprotein, APOE is fundamentally involved in lipid transport and cholesterol homeostasis, and its role in metabolic reprogramming has been increasingly linked to the modulation of the tumor immunosuppressive niche. Further computational interrogation suggested that the *APOE* protein may function as a receptor, engaging with the product of *NCF1*—expressed in CD8⁺ Tex cells—as a potential ligand. NCF1 (also known as p47phox) is a critical regulatory subunit of the NADPH oxidase complex, serving as a primary source of reactive oxygen species (ROS) that can drive chronic inflammatory signaling and metabolic stress in T cells, ultimately promoting functional exhaustion. This interaction hints at a novel *APOE*-*NCF1* axis that may contribute to tumor progression and the remodeling of an immunosuppressive microenvironment. Finally, leveraging these insights into potential mechanisms, we aim to derive and validate molecular biomarkers with translational potential, ultimately constructing a clinically actionable risk stratification framework.

## Materials and methods

### Data acquisition and preprocessing

All statistical analyses and data visualizations were conducted within the R computing environment (v4.4.0). We constructed an integrated cellular atlas comprising paracancerous, primary, and metastatic samples by acquiring single-cell RNA sequencing (scRNA-seq) data from the Gene Expression Omnibus (GEO) repository under accession number GSE60542 (https://www.ncbi.nlm.nih.gov/geo/query/acc.cgi?%20acc=GSE60542 ) and spatial transcriptomics (ST) data under accession number GSE250521 (https://www.ncbi.nlm.nih.gov/geo/query/acc.cgi?%20acc=GSE250521 ). The transcriptomic training cohort was derived from The Cancer Genome Atlas (TCGA) program, with data accessed through the Genomic Data Commons (GDC) Data Portal (https://portal.gdc.cancer.gov/ ) under project identifier TCGA-THCA, containing RNA-seq expression matrices and complete clinical follow-up data. External validation for bulk RNA-seq analysis was performed using the GSE275666 dataset (https://www.ncbi.nlm.nih.gov/geo/query/acc.cgi?%20acc=GSE275666 ),and All data were accessed and analyzed in compliance with the respective database terms of use and the Declaration of Helsinki principles for research using publicly available data.For bulk RNA-seq data, raw count matrices were transformed into Transcripts Per Million (TPM) for normalization. To ensure comparability across disparate datasets, batch effects were corrected using the ComBat algorithm from the sva package (v3.54.0). All data are publicly accessible and fully released.

### Single-cell data processing and cell type annotation

The scRNA-seq processing pipeline utilized the Seurat package (v5.4.0). Stringent quality control measures were enforced, excluding low-quality cells defined by mitochondrial gene expression exceeding 20% or a detected gene count below 200. Following data normalization via the LogNormalize method, highly variable genes were identified using the FindVariableFeatures function. To mitigate batch effects across samples, the Harmony integration algorithm (v1.2.4) was employed. Dimensionality reduction was achieved through Principal Component Analysis (PCA), with the top 20 principal components selected for visualization via Uniform Manifold Approximation and Projection (UMAP). Cell clustering was executed using the FindNeighbors and FindClusters functions. A resolution of 0.3 was selected, resulting in 22 clusters. Based on canonical marker expression, cells were annotated into nine major lineages: thyroid follicular epithelial cells (TG, TPO, EPCAM, KRT7, KRT8, KRT18, KRT19), T/NK cells (CD3E, CD3D), B cells (CD79A, CD79B), plasma cells (MZB1, IGHG1), myeloid cells (CD14, LYZ), dendritic cells (CLEC4C, LILRA4), fibroblasts (COL1A1, COL1A2), endothelial cells (PECAM1, VWF), and proliferating cells (MKI67, TOP2A).

### Multidimensional characterization of tumor heterogeneity

To resolve tumor heterogeneity, we isolated the epithelial subset and applied Non-Negative Matrix Factorization using the geneNMF package (v0.9.2), identifying molecularly distinct subpopulations, including the Trem-associated T01 subset. Genomic instability was assessed using the infercnv package (v1.22.0) to estimate copy number variations (CNV). Using microenvironmental immune cells as a normal reference baseline, totalCNV scores were calculated for each tumor cell; elevated scores confirmed the malignant clonal nature of specific subsets. Differentiation states were quantified using the CytoTRACE2 algorithm (v1.1.0). This method calculates stemness scores based on transcriptional diversity, where a score of 1 indicates maximum undifferentiated potential, revealing the developmental plasticity of tumor subpopulations. Developmental trajectories were reconstructed using Monocle 2 (v2.34.0). Dimensionality reduction via DDRTree modeled the dynamic pseudotime evolution from normal thyroid epithelium to malignant states.

### Functional enrichment and pathway inference

To elucidate the biological functions and signaling pathways associated with differentially expressed genes (DEGs) and specific cell modules, functional enrichment analysis was conducted using the clusterProfiler package (v4.14.6). Pathway activity was analyzed using the fgsea package (v1.32.4). Gene Set Enrichment Analysis (GSEA) focused on KEGG and Hallmark gene sets, with a significance threshold of *P* < 0.05, revealing significant enrichment of Hypoxia, Glycolysis, and PD-1/PD-L1 checkpoint pathways in the T01 subpopulation.

### High-resolution T cell subtyping

T/NK cell subsets were extracted and re-clustered following the same workflow as described above. A resolution of 0.4 was selected, resulting in 12 clusters. Based on distinct gene signatures, 11 subclusters were resolved: CD4 + follicular helper T cells (CD4), CD4 + Th17 cells (RORC, CCR6), CD4 + naive T cells (CCR7), CD4 + regulatory T cells (FOXP3, IL2RA), CD8 + exhausted T cells (Tex; CD8A, PDCD1, LAG3), CD8 + effector T cells (GZMK), CD8 + effector memory T cells (GZMK, CXCR5), CD8 + memory T cells (CXCR4), CD8 + naive T cells (CCR7, LEF1), CD8 + tissue-resident memory T cells (ZNF683, CD52), and NK cells (GZMB, FCGR3A, NCAM1). We prioritized the CD8 + Tex subpopulation, stratifying it into NCF1^high and NCF1^low groups based on gene expression. GSEA subsequently revealed a strong association between high NCF1 expression and immunosuppressive pathways, specifically PD-1 and TGF-β signaling.

### Cell-cell communication analysis

Intercellular communication networks were inferred using the CellChat package (v1.6.1).Ligand–receptor interactions were inferred using the CellChatDB.human database.Only interactions with *p* < 0.05 were considered significant. We quantified interaction strength by calculating the communication probability between ligand-receptor pairs, specifically targeting interactions between Trem tumor cells and CD8 + Tex cells. To delineate specific molecular regulatory mechanisms, the NicheNet algorithm (nichenetr v2.2.1.1) was used to predict ligand-target links. With Trem tumor cells designated as senders and CD8 + Tex cells as receivers, we identified APOE as the upstream ligand driving the regulation of the downstream target NCF1, establishing the APOE-NCF1 interaction axis.

### Spatial transcriptomics validation

Spatial transcriptomics data were utilized to validate scRNA-seq-inferred interactions. We mapped cell types defined by scRNA-seq to spatial locations using the deconvolution algorithm within Seurat (v5.4.0). Expression levels of APOE and NCF1 were binarized, defining the top 20th percentile as high-expression regions. We then calculated the spatial co-localization frequency and Euclidean distance between Trem cells and CD8 + Tex cells, as well as between APOE^high and NCF1^high spots. Statistical analysis confirmed that these spatial distances were significantly reduced in tumor tissues compared to adjacent normal tissues, providing physical evidence for the proposed interaction.

### Weighted gene co-expression network analysis

Gene co-expression networks were constructed for tumor and T/NK cells using the WGCNA package (v1.73). Scale-free networks were established by determining the optimal soft-thresholding power. Module eigengenes were then correlated with CD8 + Tex phenotypes and clinical traits to identify key gene modules associated with immune exhaustion and tumor progression.

### Prognostic model construction and machine learning screening

We constructed gene co-expression networks using the hdWGCNA framework to identify modules of co-expressed genes, capturing systems-level gene programs beyond individual differentially expressed genes.HDWGCNA was chosen to identify systems-level gene modules, which can reveal regulatory programs and pathway-level signals beyond lists of DEGs. Through hdWGCNA, the CD8⁺Tex_NEW4 module most correlated with CD8⁺Tex and the Salmon module most correlated with T01 (Trem) tumor cells were identified, respectively. A consensus gene set was then obtained by intersecting the key module genes from WGCNA with the differentially expressed genes from the GSE60542 cohort. Gene modules were identified using blockwiseModules with: minModuleSize = 30;mergeCutHeight = 0.25;deepSplit = 2.Prognostic candidates were initially filtered via univariate Cox regression (survival v3.8-6), followed by feature dimensionality reduction using LASSO regression (glmnet v4.1-10). To construct a robust prognostic model, we evaluated 101 machine learning algorithm combinations (incorporating packages such as randomForestSRC v3.4.5) within the training set and selected the optimal framework via cross-validation. A final multivariate Cox proportional hazards model and a nomogram were generated. Model performance was systematically assessed using Kaplan-Meier survival analysis and time-dependent ROC curves across both training and independent validation cohorts, confirming the efficacy of the risk score in stratifying patient prognosis.

## Result

### Construction of a single-cell atlas for thyroid carcinoma, batch effect correction, and annotation of major cell types

**Fig. 1:**
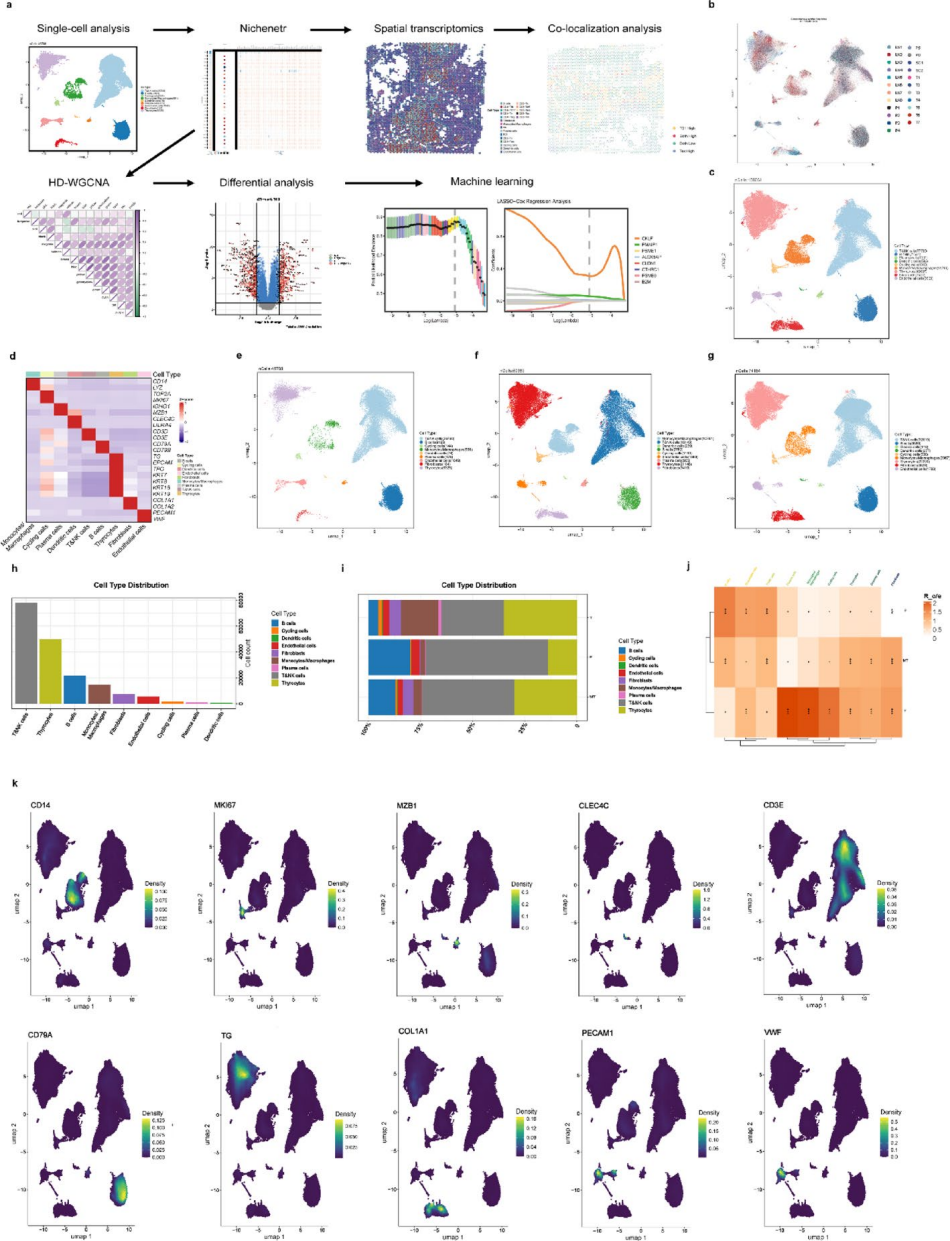
Integrated single-cell transcriptomic analysis, atlas construction, and cellular compositional differences across tissue origins in thyroid carcinoma. **a **Schematic overview of the overall analytical workflow **b** Visualization assessing the effectiveness of batch effect correction in the integrated single-cell dataset **c** UMAP dimensionality reduction plot visualizing all cells **d** Expression distribution of key marker genes used for annotating the major cell types **e–g** UMAP plots stratified by tissue origin: adjacent normal tissue (P) **e**, primary tumor (T) **f**, and metastatic tumor (MT) (**g**) **h** Overall abundance distribution of each major cell type across the entire cohort **i** Comparison of the relative abundance of each cell type across different tissue origins (P, T, MT) **j** Tissue preference analysis (Ro/effect score) evaluating the enrichment tendency of each cell population in P, T, and MT groups **k** Validation of representative marker gene expression across the annotated cell types

This study integrated single-cell transcriptomic data from thyroid carcinoma samples, performed quality control and batch effect correction, constructed a global cellular atlas, and completed cell type annotation. Subsequent analyses investigated cellular compositional differences and tissue preferences across different tissue origins—adjacent normal tissue (P), primary tumor (T), and metastatic tumor (MT)—providing a critical foundation for subsequent efforts. These include focusing on key cellular populations and their interactions, validating findings with spatial transcriptomics, and utilizing machine learning to identify prototypical disease biomarkers and construct a prognostic model (Fig. 1a).

We first assessed and corrected for batch effects in the integrated dataset. Following correction, cells from different batches showed substantial intermixing in the low-dimensional space, indicating the integration strategy effectively minimized technical variation, thereby ensuring the reliability of downstream cross-group comparisons (Fig. 1b). The integrated UMAP visualization revealed that the thyroid carcinoma microenvironment is composed of diverse immune and non-immune cell populations. All cells formed several distinct clusters within the UMAP space (Fig. 1c). A total of 180,261 cellswere classified into nine major cell types based on canonical marker gene expression, including: T and NK cells: 77,760 cells (43.1%);Thyrocytes: 49,697 cells (27.6%);B cells: 21,534 cells (11.9%);Monocytes/Macrophages: 14,719 cells (8.2%);Fibroblasts: 7,422 cells (4.1%);Endothelial cells: 5,523 cells (3.1%);Cycling cells: 1,813 cells (1.0%);Plasma cells: 1,201 cells (0.7%);Dendritic cells: 592 cells (0.3%).These results demonstrate that immune cells and thyroid epithelial cells represent the dominant cellular components of the tumor microenvironment (Fig. 1d).

The spatial distribution of cells from different tissue origins within the UMAP showed discernible variation, suggesting ecosystem remodeling accompanies disease progression. Stratified visualization of cells from adjacent normal (P) (Fig. 1e), primary tumor (T) (Fig. 1f), and metastatic tumor (MT) (Fig. 1g) tissues revealed differences in the proportion and distribution across multiple clusters. This indicates dynamic shifts in immune infiltration and stromal/epithelial components during tumorigenesis and metastasis. Cellular abundance analysis quantified the compositional differences among groups, revealing T&NK cells as the most abundant population overall (Fig. 1h). Further group-specific analysis showed: B cells were most abundant in the P group, followed by MT, and lowest in T; T&NK cells followed a similar pattern, highest in P, then MT, and lowest in T. In contrast, Thyrocytes were most abundant in the MT group, followed by T, and lowest in P. These results suggest that adjacent normal tissue possesses a higher proportion of lymphocytes (B cells and T/NK cells), while metastatic foci exhibit a greater proportion of thyroid-derived cells (Thyrocytes), reflecting distinct microenvironmental compositions at different disease stages (Fig. 1i).

Tissue preference analysis further delineated the enrichment tendencies of specific cell populations for different tissue stages. Based on the Ro/effect score, B cells showed the most pronounced preference for the P group; Fibroblasts exhibited a preference for the MT group; and Plasma cells displayed a more prominent preference for the T group. These preference results corroborate the abundance differences, suggesting that distinct immune and stromal cell populations may assume differential functional roles within the tumor microenvironment during the primary and metastatic stages. Collectively, this analysis provides a comprehensive atlas for subsequently pinpointing key cellular subpopulations and their potential interactions (Fig. 1j).

The expression patterns of key markers further validated the annotation accuracy, with distinct cell types exhibiting the expected specific expression: *CD14* was highly expressed in Monocytes/Macrophages, *MKI67* marked Cycling cells, *MZB1* marked Plasma cells, *CLEC4C* marked Dendritic cells, *CD3E* marked T&NK cells, *CD79A* marked B cells, *TG* marked Thyrocytes, *COL1A1* marked Fibroblasts, while *PECAM1* and *VWF* marked Endothelial cells, collectively supporting the robustness of the cell type assignments (Fig. 1k, Supplementary Fig. S2). To further ensure the accuracy of thyrocyte annotation, we validated the expression of several additional canonical thyroid and epithelial markers, including *TPO*,* KRT7*,* KRT8*,* KRT18*, and *KRT19*. These markers exhibited highly specific and robust expression patterns within the Thyrocyte cluster (Supplementary Figure S1), confirming our initial assignment.

### Subpopulation classification and functional phenotypic characterization of thyroid tumor cells

**Fig. 2:**
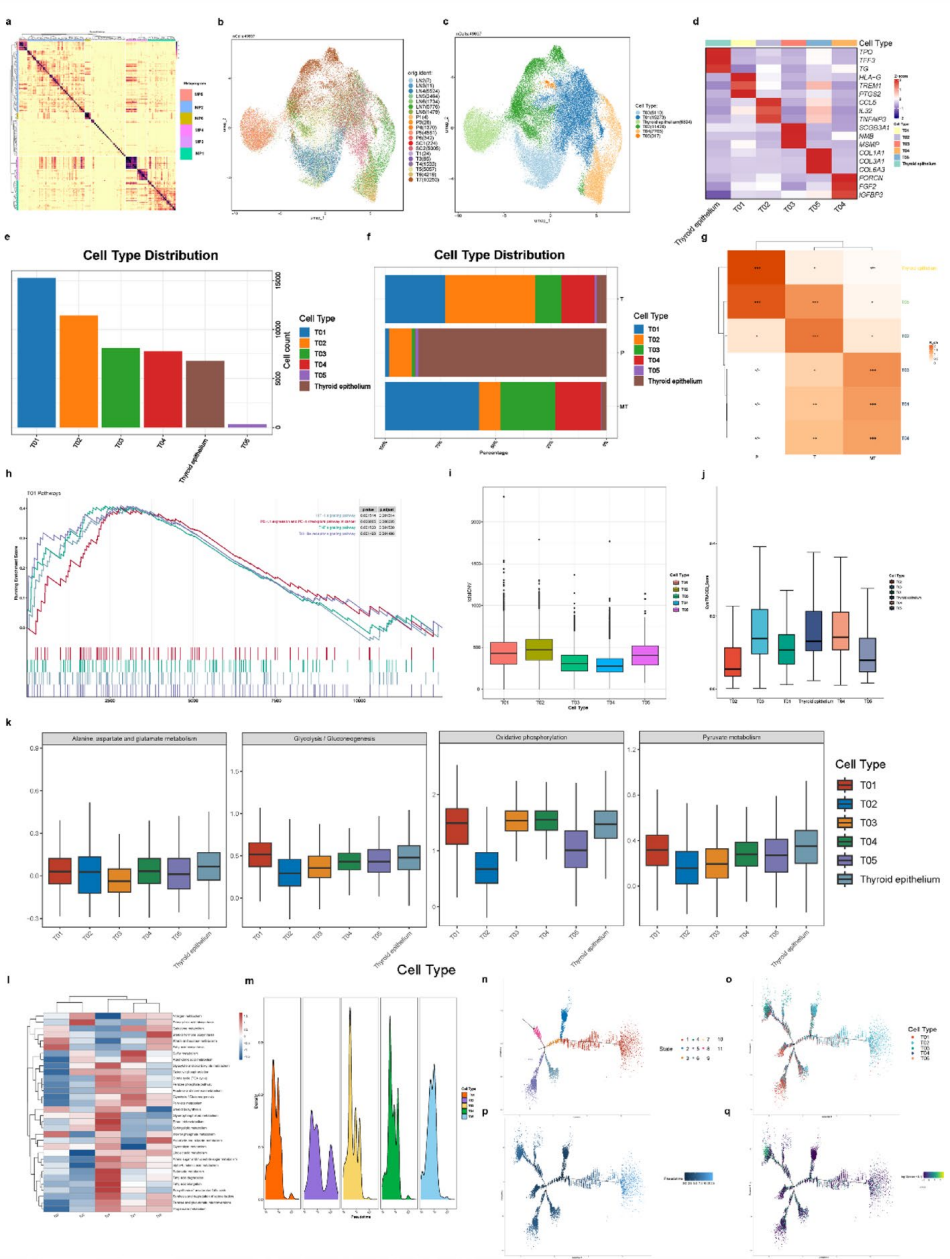
Subpopulation classification, tissue preference, and functional phenotypic characterization of thyroid tumor cells. **a** Heatmap of geneNMF clustering **b** Assessment of batch correction for the tumor/epithelial cell subset **c** UMAP clustering plot of tumor/epithelial cell subpopulations **d** Expression of representative markers for each subcluster **e** Abundance distribution of each subcluster across all samples **f** Relative abundance of each subcluster across different tissue origins (P, T, MT) **g** Tissue preference (Ro/effect score) analysis **h** GSEA results highlighting enriched pathways in the T01 subcluster **i** Total copy number variation (totalCNV) analysis across subclusters (j) CytoTRACE2 scores (evaluating developmental/undifferentiated features) **k** Comparison of metabolic pathway activities **l** Overall pathway enrichment results **m–q** Pseudotime trajectory analysis illustrating the dynamic evolution of tumor/epithelial cells towards distinct functional states/subpopulations

To elucidate the intrinsic heterogeneity of thyroid tumor cells, we performed subclustering and functional phenotypic analysis on the integrated epithelial/tumor cell subset. Initially, geneNMF was employed to factorize the expression matrix, with cluster heatmaps assisting in defining cell subpopulations (Fig. 2a). Evaluation of batch correction demonstrated effective mixing of cells from different batches in the low-dimensional space (Fig. 2b). Unsupervised clustering, validated by marker gene expression, identified several tumor/epithelial cell subpopulations. A total of 49,697 thyroid epithelial cells included Thyroid epithelium 6,804 cells(markers: *TPO*, *TFF3*) and five subclusters designated T01–T05: T01 15,273 cells (Trem, markers: *HLA-G*, *PTGS2*), T02 11,428 cells (markers: *CCL5*, *TNFAIP3*), T03 8,110 cells (markers: *SCGB3A1*, *NMB*, *MSMP*), T04 7,765 cells (markers: *PORCN*, *FGF2*), and T05 317 cells (stromal/mesenchymal-like, markers: *COL1A1*, *COL3A1*, *COL6A3*) (Fig. 2c–d).

Abundance analysis revealed significant differences in the distribution of these subpopulations both overall and across groups: T01 was most abundant in the MT group, followed by the T group, and lowest in the P group; T02 peaked in the T group, with P and MT groups showing similar levels; T03 and T04 were most enriched in the MT group; Thyroid epithelium was most prevalent in the P group (Fig. 2e–f). Tissue preference analysis further indicated that Thyroid epithelium exhibited the strongest preference for the P group; T01 and T02 showed a stronger tendency towards the T group; while T03 displayed the highest preference for the MT group (Fig. 2g).

Functional enrichment and malignant phenotype-related analyses underscored the potential oncogenic/metastatic characteristics of T01. Gene Set Enrichment Analysis (GSEA) of T01 highlighted pathways including the HIF-1 signaling pathway, PD-L1 expression and PD-1 checkpoint pathway in cancer, TNF signaling pathway, and Toll-like receptor signaling pathway, suggesting this subpopulation exhibits features of stress adaptation, immune regulation, and pro-inflammatory pathway activation (Fig. 2h). Copy number variation (totalCNV) analysis indicated a relatively high score for T01. This metric is commonly used to characterize the degree of genomic instability/tumorigenicity; the elevated totalCNV in T01 supports its phenotype being closer to that of a tumor cell (i.e., more likely a clonal or malignancy-associated population). Therefore, within this study, T01 can be considered a key tumor cell subpopulation closely associated with tumor progression (Fig. 2i).

Analyses of metabolic and developmental states provided further support for the highly active/malignant phenotype of T01. CytoTRACE2 scoring suggested that T01 possesses higher “undifferentiated/developmental potential” or stem-like features (Fig. 2j). Enrichment of metabolic pathways revealed that T01 exhibited the highest activity levels in Alanine, aspartate and glutamate metabolism, Glycolysis/Gluconeogenesis, Oxidative phosphorylation, and Pyruvate metabolism. Such metabolic reprogramming may promote cell proliferation, hypoxia tolerance, and an invasive phenotype, thereby contributing to the malignancy of T01 (Fig. 2k). Overall pathway enrichment and pseudotime trajectory analysis delineated a dynamic transition from a relatively “normal/epithelial” phenotype towards tumor-characteristic subpopulations, particularly T01. This establishes a cellular foundation for subsequent mechanistic investigations into the interactions between T01 and immune-exhausted T cells (Fig. 2l–q).

### T cell subpopulation analysis reveals strong communication between CD8⁺ Tex and T01 (Trem tumor cells) and implicates an apoe–ncf1 interaction axis

**Fig. 3:**
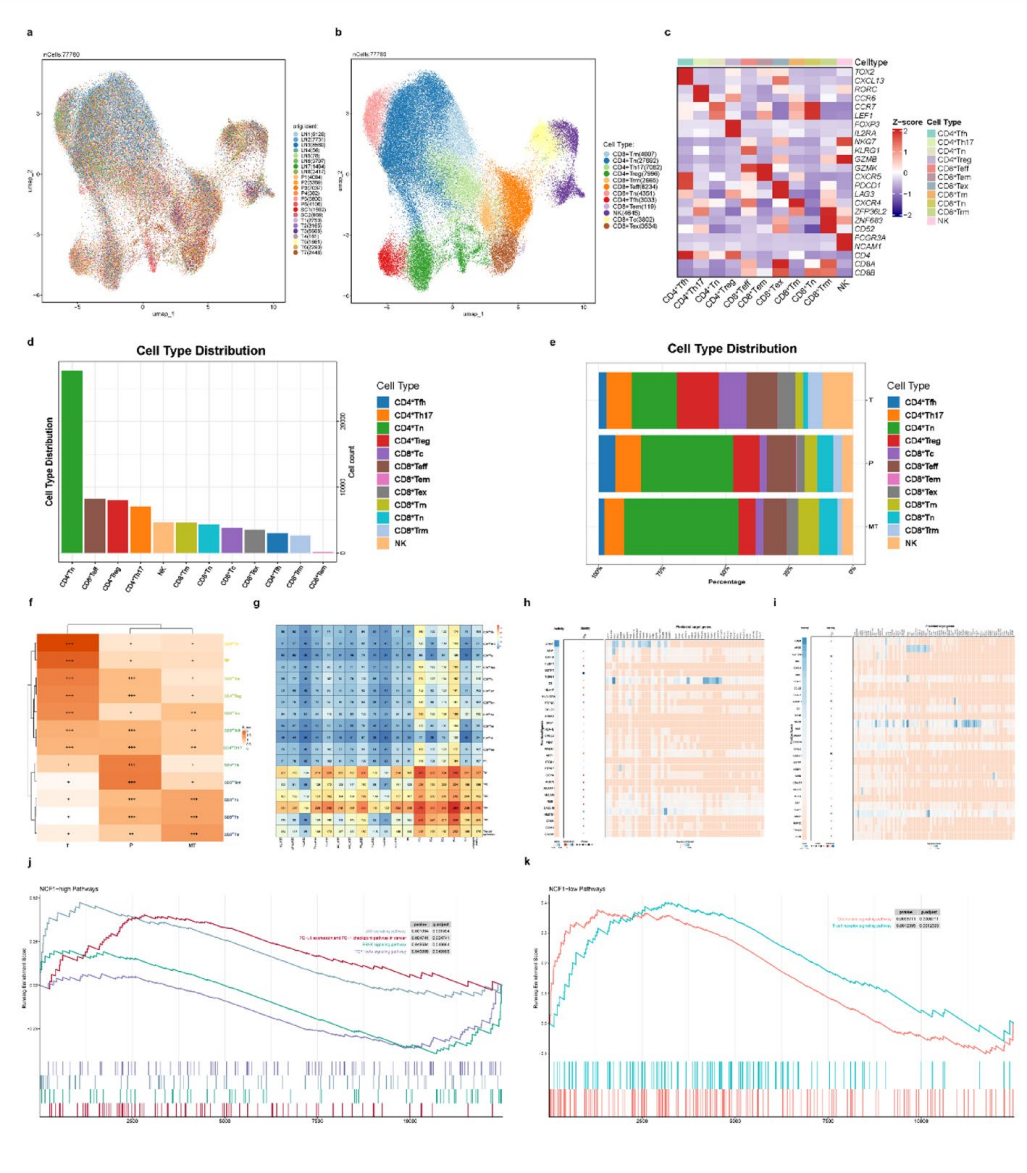
T cell subpopulation classification, tissue preference, and molecular communication clues between Trem and CD8⁺ Tex. **a** Low-dimensional visualization of the integrated T cell subset post-batch correction, assessing integration quality **b** UMAP clustering plot of T cell subpopulations **c** Expression of representative markers for each T cell subpopulation **d** Overall abundance distribution of each T cell subpopulation **e** Comparison of the relative abundance of each subpopulation across tissue origins **f** Results of tissue preference (Ro/effect score) analysis **g** Evaluation of intercellular communication using CellChat **h–i** NicheNet predictions of potential ligand-target gene relationships between different comparison groups **j–k** GSEA results for CD8⁺ Tex cells stratified into high and low NCF1 expression groups

To elucidate the heterogeneity of T cells within the thyroid carcinoma tumor microenvironment, we performed reclustering and annotation on the T cell subset from the integrated data. After batch correction, T cells from all sample origins showed effective mixing in the low-dimensional space, confirming successful mitigation of batch effects (Fig. 3a). UMAP visualization based on unsupervised clustering revealed multiple, well-separated T cell subpopulations. Annotation guided by marker gene expression identified the following subsets: CD4⁺ Tfh (marker: *CD4*), CD4⁺ Th17 (markers: *RORC*, *CCR6*), CD4⁺ Tn (marker: *CCR7*), CD4⁺ Treg (markers: *FOXP3*, *IL2RA*), CD8⁺ Teff (marker: *GZMK*), CD8⁺ Tem (markers: *GZMK*, *CXCR5*), CD8⁺ Tex (markers: *CD8A*, *PDCD1*, *LAG3*), CD8⁺ Tm (marker: *CXCR4*), CD8⁺ Tn (markers: *CCR7*, *LEF1*), CD8⁺ Trm (markers: *ZFP36L2*, *ZNF683*, *CD52*), and NK cells (markers: *GZMB*, *FCGR3A*, *NCAM1*). Among these, CD8⁺ Tex exhibited canonical exhaustion markers (*PDCD1*, *LAG3*) and became the primary focus for subsequent investigation .Within the 77,760 T and NK cells, twelve transcriptionally distinct subpopulations were identified, including: CD4⁺ Tn: 27,692 cells (35.6%);CD8⁺ Teff: 8,234 cells (10.6%);CD4⁺ Treg: 7,996 cells (10.3%);CD4⁺ Th17: 7,082 cells (9.1%);CD8⁺ Tm: 4,607 cells (5.9%);NK cells: 4,645 cells (6.0%);CD8⁺ Tn: 4,351 cells (5.6%);CD8⁺ Tc: 3,802 cells (4.9%);CD8⁺ Tex: 3,534 cells (4.5%);CD4⁺ Tfh: 3,033 cells (3.9%);CD8⁺ Trm: 2,665 cells (3.4%);CD8⁺ Tem: 119 cells (0.15%)(Fig. 3b–c).

Quantitative analysis of subpopulation abundance and comparison across tissue origins revealed significant compositional differences in T cell subsets at distinct disease stages (Fig. 3d–e). Tissue preference analysis indicated that CD8⁺ Tem and CD4⁺ Tfh showed the strongest preference for the P group; CD8⁺ Tm was preferentially enriched in the MT group; while CD8⁺ Tex demonstrated a clearer preference for the T group (Fig. 3f). These differences suggest dynamic remodeling of the T cell repertoire and functional states during disease progression.

Cell communication analysis uncovered robust interactions between the tumor cell subcluster T01 (subsequently defined as the Trem tumor cell group) and exhausted CD8⁺ Tex. Assessment of interaction strength using CellChat indicated significantly elevated communication between Trem and CD8⁺ Tex, characterized by bidirectional enhanced signaling (i.e., high interaction strength for both Trem→CD8⁺ Tex and CD8⁺ Tex→Trem). This suggests these populations may mutually influence the tumor microenvironmental state by acting as both signal sources and targets for each other (Fig. 3g).

To identify potential ligand–receptor/target gene axes at the molecular level, we employed NicheNet. When comparing adjacent normal tissue to primary tumor (Fig. 3h) and primary tumor to metastatic tumor (Fig. 3i), NicheNet consistently ranked *APOE*—expressed by T01—among the top candidate ligands (*APOE* was Top1 in Fig. 3h and Top2 in Fig. 3i). Concurrently, *NCF1* in CD8⁺ Tex was identified as a potential downstream target gene, supporting *APOE* (from Trem)—*NCF1* (from CD8⁺ Tex) as a candidate intercellular interaction axis. This axis provides a precise molecular target for subsequent spatial validation and functional dissection.

Based on *NCF1* expression levels within the CD8⁺ Tex population, we stratified these cells into *NCF1*-high and *NCF1*-low groups and performed GSEA. The results showed that the *NCF1*-high group was enriched for pathways including the p53 signaling pathway, *PD-L1* expression and *PD-1* checkpoint pathway in cancer, PPAR signaling pathway, and TGF-beta signaling pathway. In contrast, the *NCF1*-low group showed relative enrichment for the chemokine signaling pathway and T cell receptor signaling pathway (Fig. 3j–k). These differential enrichment patterns suggest that a high *NCF1* expression state may be associated with inhibitory/tolerogenic pathways (e.g., PD-1/PD-L1, TGF-β), thereby coupling with CD8⁺ Tex exhaustion and the tumor immunosuppressive microenvironment.

### Spatial co-localization validates the APOE–NCF1 interaction axis and spatial proximity between Trem and CD8⁺ Tex

**Fig. 4:**
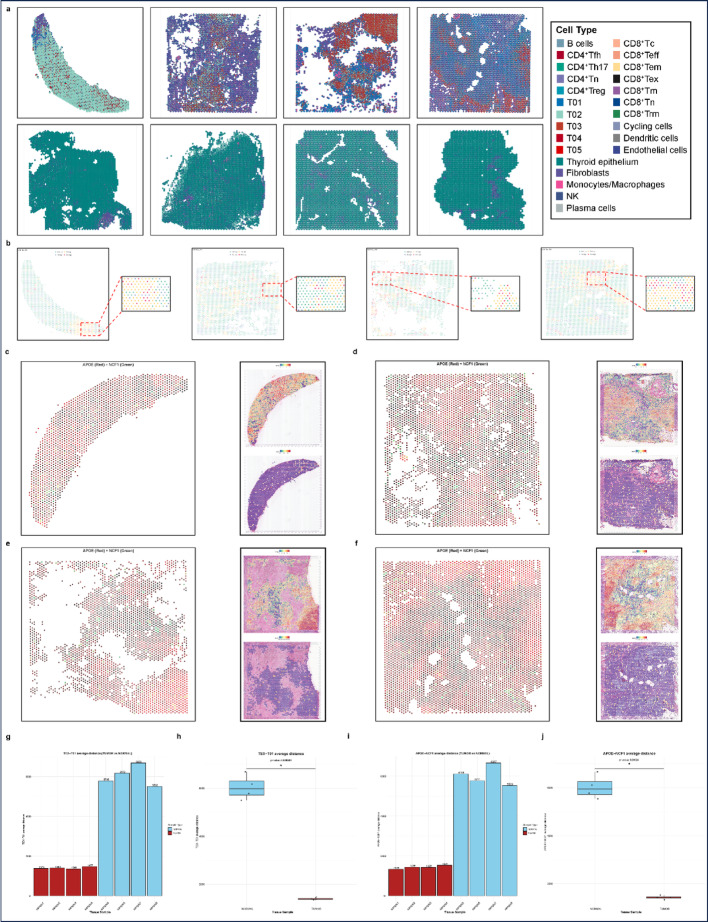
Spatial transcriptomic validation: spatial co-localization of Trem and CD8⁺ Tex and the *APOE–NCF1* Interaction Axis. **a** Results of deconvolution analysis on ST sections **b **Schematic of strategies for co-localization analysis **c–f** Expression distribution, binarized high-expression zones, and overlap regions for *APOE* and *NCF1* in representative tumor sections **g–h **Comparison of Euclidean distances between CD8⁺ Tex and Trem in normal versus tumor tissues **i–j ** Analysis of Euclidean distances between spatial locations expressing *APOE* and those expressing *NCF1*

To validate the Trem–CD8⁺ Tex interaction axis and the spatial relationships of the associated ligand/receptor pair identified in single-cell analyses, we performed deconvolution and co-localization analyses on spatial transcriptomics (ST) tissue sections. Deconvolution results, resolving cellular composition within each ST spot into relative abundances of cell types, are presented: the upper four panels show ST sections from the T group (primary tumor), and the lower four panels show sections from the P group (adjacent normal). Enriched tumor cell subpopulations (Trem) and exhausted T cell clusters (CD8⁺ Tex) were detectable in tumor sections, while lymphocyte distribution was more dispersed in normal adjacent tissue sections (Fig. 4a).

Subsequently, for each cell type (or gene), the expression/score in each spatial location was ranked within its sample. Positions falling within the top 20% were defined as “high-expression/high-enrichment” (assigned a value of 1), while the remaining 80% were designated “low-expression/low-enrichment” (assigned a value of 0). Based on this binarization, the co-localization frequency/ratio of Trem and CD8⁺ Tex across sections was calculated and compared between samples. The results demonstrated that their spatial overlap was significantly higher than expected by random chance (Fig. 4b).

The spatial co-localization of *APOE* (primarily originating from Trem) and *NCF1* (primarily from CD8⁺ Tex) is presented for representative tumor sections, displaying their expression distribution, binarized high-expression zones, and overlapping regions. Quantitative analysis confirmed a high spatial co-localization rate between *APOE* high-expression and *NCF1* high-expression zones. This supports the observability of the *APOE*→*NCF1* communication axis, inferred from single-cell data, at the tissue spatial level (Fig. 4c–f).

We further compared the spatial distance distribution between CD8⁺ Tex and Trem in normal versus tumor tissues. The results show that the average Euclidean distance between CD8⁺ Tex and Trem was significantly smaller in the tumor microenvironment than the distance between corresponding cell types in normal tissue, indicating a closer spatial neighbor relationship in the tumor context (Fig. 4g–h). Similarly, calculating the spatial distance between *APOE*-expressing T01 locations and *NCF1*-expressing CD8⁺ Tex locations revealed a significantly shorter distance in tumor sections compared to normal tissue. This further supports the spatial proximity of the *APOE*–*NCF1* pair and the feasibility of local intercellular signaling (Fig. 4i–j).

### WGCNA identifies pivotal gene modules in thyroid tumor cells and T/NK Cells

To systematically identify gene modules associated with the functional states of tumor cells and immune cell populations, we performed Weighted Gene Co-expression Network Analysis (WGCNA) separately on the thyroid carcinoma epithelial/tumor cell subset and the T&NK cell subset.

**Fig. 5:**
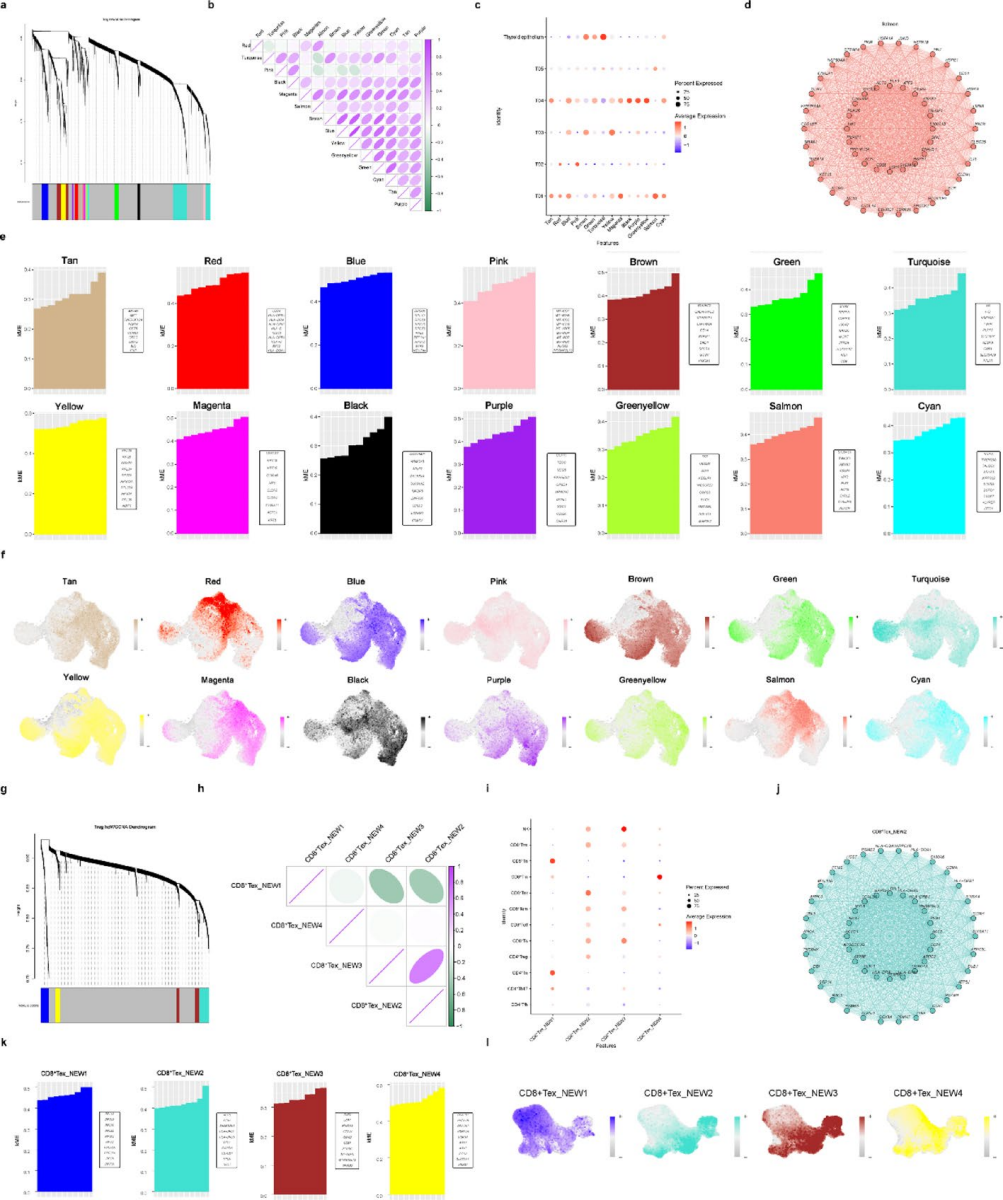
WGCNA-Based Modular Gene Networks Reveal Key Gene Motifs in Tumor Cells and T&NK Cells. **a** Distribution of WGCNA modules constructed from the thyroid carcinoma epithelial/tumor cell subset **b–c** Heatmap/scatter plots of module-trait correlations **d** Key module identified through integrated correlation and functional annotation **e **Top ten representative genes for each of the 14 modules (Thyroid carcinoma cell modules) **f** Expression profiles of module genes across thyroid carcinoma tissue samples **g** Distribution of WGCNA modules constructed from the T&NK cell subset **h–i** Results of module-trait correlation analysis for T&NK modules **j** The CD8^+^_NEW2 module and its member genes, identified as the key module for T&NK cells **k** Top ten representative genes for each of the four T&NK modules **l** Expression distribution of T&NK module genes within the T&NK cell population

Within the thyroid carcinoma cell data, WGCNA partitioned the transcriptome into 14 distinct co-expression modules (designated Tan, Red, Blue, Pink, Brown, Green, Turquoise, Yellow, Magenta, Black, Purple, Greenyellow, Salmon, Cyan) (Fig. 5a). Module-trait correlation analysis was conducted to evaluate the associations of each module with phenotypic/clinical features (Fig. 5b–c). Based on a synthesis of these correlation results and functional annotation, the Salmon module and its member genes were identified as a pivotal gene set within the tumor cell population (Fig. 5d). The figure also presents the top ten representative genes for each module (Fig. 5e) and depicts the expression profile characteristics of module genes across thyroid carcinoma tissue samples (Fig. 5f).

In the T&NK cell data, WGCNA delineated four co-expression modules (CD8+_NEW1, CD8+_NEW2, CD8+_NEW3, CD8+_NEW4) (Fig. 5g). Subsequent module-trait correlation analysis (Fig. 5h–i) enabled the screening of modules significantly correlated with CD8⁺ Tex functional states or clinical characteristics. Among these, the CD8+_NEW2 module was established as the key gene module for the T&NK population (Fig. 5j). The figure lists the top ten representative genes for each of the four modules (Fig. 5k) and illustrates the expression distribution of module genes within the T&NK cell population (Fig. 5l).

### Integration of WGCNA and external cohort data for screening and construction of a thyroid carcinoma prognostic model

**Fig. 6:**
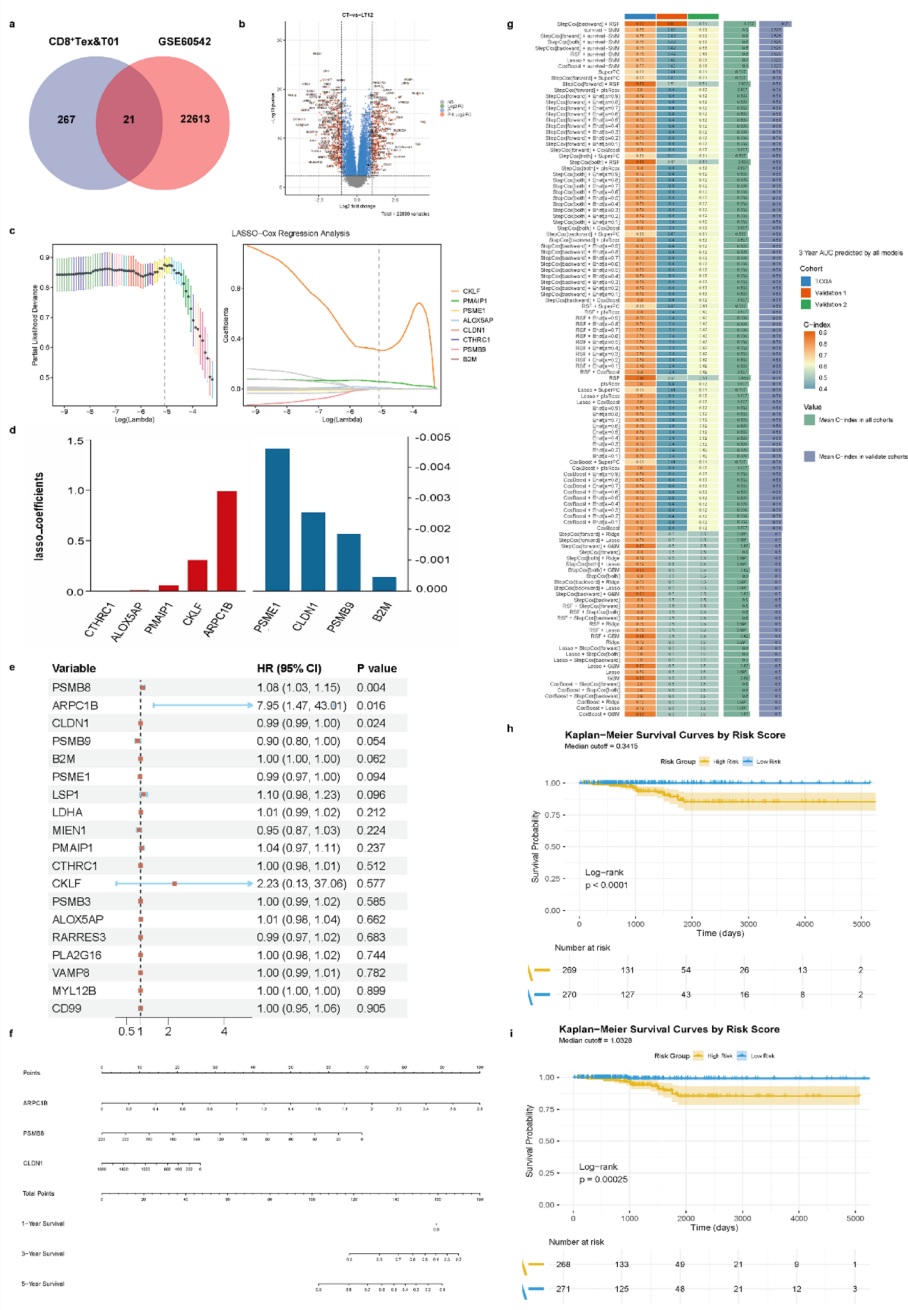
Integrated screening using WGCNA and external cohorts, and construction of a thyroid carcinoma prognostic model **a** Venn diagram illustrating the identification of 21 candidate genes **b** Volcano plot of differential expression analysis based on the GSE60542 cohort **c** LASSO regression analysis for screening core genes **d** Regression coefficients of the core genes in the LASSO model **e** Multivariate Cox regression analysis identifying independent prognostic genes **f** Nomogram constructed based on the three-gene multivariate Cox model **g** Performance comparison of prognostic models built from 101 machine learning algorithms using the prognostic gene set **h** Kaplan–Meier survival curves stratifying patients into high/low-risk groups based on the multivariate Cox risk score **i** Kaplan–Meier survival curves stratifying patients into high/low-risk groups based on the LASSO risk score

We first integrated the key module genes identified by the aforementioned WGCNA in the T01 (Trem) and CD8⁺ Tex subsets, taking their union. Subsequent intersection analysis via Venn diagram with the gene set from the external cohort GSE60542 yielded 21 candidate genes for downstream screening (Fig. 6a). Differential expression analysis between normal thyroid and thyroid carcinoma samples in GSE60542 was performed, with a volcano plot illustrating significant expression differences for multiple genes between tumor and normal tissues (Fig. 6b).

Subsequent univariate Cox regression analysis on the 21 candidate genes identified 19 genes associated with prognosis. Dimensionality reduction was then applied using LASSO regression, resulting in the selection of 8 core genes (*CKLF*, *PMAIP1*, *PSME1*, *ALOX5AP*, *CLDN1*, *CTHRC1*, *PSMB9*, *B2M*) along with their corresponding LASSO coefficients (Fig. 6c–d). To identify independent prognostic factors, the 19 genes from the univariate screening were subjected to multivariate Cox regression analysis. This final step established *ARPC1B*, *PMSB8*, and *CLDN1* as independent prognostic genes (Fig. 6e). A nomogram based on these three genes was constructed for individualized survival risk prediction (Fig. 6f).

Furthermore, clinical samples from the TCGA thyroid carcinoma database were randomly partitioned into three subsets (60%, 20%, 20%). The 60% subset served as the training set for machine learning model development, while the remaining two subsets were used as validation sets to test model performance. Prognostic models based on the aforementioned genes were constructed and compared across 101 distinct machine learning algorithm combinations. Among these, the StepCox (backward) + RSF (Random Survival Forest) combination demonstrated optimal performance in 3-year survival prediction, achieving AUC values of 0.88 in TCGA, 0.91 in Validation1, and 0.51 in Validation2 (Fig. 6g).

Finally, risk scores were calculated based on the multivariate Cox and LASSO models, respectively, and patients were stratified into high-risk and low-risk groups. Kaplan–Meier survival curves demonstrated that both scoring systems effectively distinguished patient populations with divergent prognoses (Fig. 6h–i), indicating that this gene signature and its associated machine learning model possess prognostic stratification potential and a degree of cross-cohort generalizability.

## Discussion

Thyroid carcinoma, particularly the differentiated subtypes, is often characterized by an overall indolent clinical course [20]. However, aggressive progression, recurrence, and distant metastasis remain significant clinical challenges [21]. A key contributor to this heterogeneity is the profound intratumoral diversity in cellular states: distinct subpopulations vary considerably in their proliferative capacity, invasive potential, metabolic reprogramming, antigen presentation, and ability to interact with immune cells [22]. The tumor cell subpopulations we identified suggest the existence of a cellular state more prone to coupling with immunosuppressive networks. This state is not necessarily defined by the highest proliferative rate but may instead remodel the local immune niche through altered cell-cell communication, metabolism, and inflammatory signaling, thereby driving CD8⁺ T cell dysfunction and facilitating sustained tumor growth. This finding underscores the possibility that in thyroid carcinoma, tumor cells are not passive recipients of immune pressure but active architects of an immunosuppressive microenvironment. It also provides direction for subsequent efforts to delineate the transcriptional regulatory networks and upstream drivers—such as hypoxia, lipid metabolism, inflammatory signaling, or specific genetic alterations—underpinning this state.

CD8⁺ exhausted T cells (CD8⁺ Tex) are a central focus in tumor immunology [23]. Diverging from the traditional effector/memory paradigm, Tex should be understood as a spectrum of states arising from persistent antigen stimulation and immunosuppressive signals: ranging from precursor/stem-like exhausted cells, which retain some proliferative potential and may be more amenable to “reinvigoration” by immunotherapy, to terminally exhausted cells with diminished cytotoxic function, higher inhibitory receptor expression, and blunted responsiveness to stimulation [24]. Our meticulous annotation of T cells, with a specific focus on CD8⁺ Tex, is significant because while the overall response rate to immunotherapy in thyroid cancer may be lower than in high mutational burden tumors, specific immune niches within certain patient subsets may still present a window for intervention [25]. By identifying the presence of Tex and their coupled relationships with neighboring cell types—such as specific tumor cell states—at single-cell resolution, we can better explain the marked interpatient heterogeneity in immune-related treatment responses and disease progression observed within the same histological type. This also lays the groundwork for future exploration of which combinations of “Tex subsets + tumor cell states” hold the greatest clinical significance.

The potential interaction we propose—with *APOE* (as a receptor) from Trem tumor cells and *NCF1* (as a ligand) from CD8⁺ Tex—serves as a key mechanistic clue promoting tumor progression. While the direct, “molecule-to-molecule” nature of this interaction requires further experimental validation, its biological framework suggests several pertinent avenues for discussion. First, it highlights the coupling of lipid metabolism and immunosuppression: *APOE* plays a crucial role in lipid transport and metabolic regulation [26]. Lipid metabolic reprogramming in tumor cells is frequently associated with the establishment of an immunosuppressive microenvironment [27]. The high expression of *APOE* in the Trem subset may indicate enhanced lipid metabolic adaptability or a propensity to influence the state of neighboring immune cells via lipid-related signaling. Second, *NCF1* is linked to ROS/inflammatory pathways: as a component of the NADPH oxidase complex, *NCF1* is involved in reactive oxygen species (ROS) generation and inflammatory signaling [28]. Elevated ROS and chronic inflammation within the TME can promote T cell dysfunction, increase metabolic stress, and help maintain the exhausted phenotype [29]. Active *NCF1*-related pathways in CD8⁺ Tex may signify a state of heightened oxidative stress and chronic stimulation, further entrenching exhaustion. Third, this hints at a potential “tumor cell state–immune exhaustion” positive feedback loop: Trem tumor cells might enhance their fitness and immunomodulatory capacity via APOE-related pathways. Concurrently, Tex cells, under oxidative stress and immunosuppressive signals, become further exhausted, leading to diminished anti-tumor effector function. This, in turn, reduces immune-mediated clearance of the Trem subset, creating a feed-forward loop that facilitates persistent tumor progression.

The identification of the *APOE-NCF1* axis provides a mechanistic bridge between tumor metabolic reprogramming and T cell dysfunction. Beyond a simple spatial co-localization, the biological implication of this interaction suggests a ‘metabolic-redox’ feedback loop within the thyroid carcinoma microenvironment. *APOE* secreted by tumor subclusters may not only facilitate lipid accumulation in the TME but also potentially trigger *NCF1*-mediated oxidative stress in infiltrating CD8^+^ T cells. High levels of ROS produced via *NCF1* are known to disrupt TCR signaling and promote the expression of inhibitory receptors like PD-1 and LAG-3, thereby locking T cells into a terminal exhaustion state.

The functional consequence of this crosstalk is the formation of a ‘shielded’ immunosuppressive niche that protects tumor cells from immune surveillance. From a clinical perspective, this suggests that the failure of current anti-PD-1 therapies in some thyroid cancer patients may be attributed to these metabolic barriers. Therefore, targeting the *APOE-NCF1* axis—perhaps by combining ROS scavengers or lipid metabolism inhibitors with traditional immune checkpoint inhibitors—represents a promising strategy to reinvigorate exhausted T cells. Future studies should focus on in vitro co-culture models to validate whether neutralizing the *APOE-NCF1* interaction can restore the cytolytic activity of Tex cells, offering a more precise foundation for personalized immunotherapy in aggressive thyroid carcinoma.

A classical critique of cell-cell communication inferred from single-cell data is that complementary gene expression does not equate to physical contact or spatial co-localization. We addressed this by using spatial transcriptomics to compare the co-localization and spatial distances between Trem and CD8⁺ Tex, finding them in closer proximity in tumor tissue compared to normal tissue. Furthermore, *APOE* (Trem) and *NCF1* (CD8⁺ Tex) showed a corresponding spatial proximity. This links “molecular interaction inference” with “tissue architectural evidence,” significantly enhancing the plausibility of the proposed axis. More importantly, spatial adjacency often implies shared microenvironmental factors (e.g., hypoxia, metabolic substrates, cytokine gradients, stromal barriers). Therefore, the Trem–Tex co-localized niche likely possesses unique microenvironmental features. Future studies integrating spatial pathway activity, cytokine distribution, or immunosuppression scores could help define a more precise “immunosuppressive spatial niche” and elucidate its relationship with invasion, recurrence, or treatment response.

Building upon candidate gene screening, we performed Cox/LASSO/multivariate analyses to construct a nomogram and evaluated the generalization performance of 101 machine learning algorithms. The StepCox (backward) + RSF combination demonstrated optimal 3-year AUC performance, with notable variation between the training and validation sets. These results suggest that the molecular network associated with the Trem–Tex niche not only provides insights into potential mechanisms but may also harbor prognostic information with translational potential.

In summary, this study leverages single-cell and spatial transcriptomic data to systematically characterize the cellular composition and functional states of the thyroid carcinoma microenvironment. It focuses on tumor cell subpopulations and the immune exhaustion axis to mine mechanistic clues and explore clinical translation. We identified and defined the Trem tumor cell subset with characteristic molecular expression and potential immunomodulatory capacity, and performed fine-grained annotation of the T cell lineage, focusing on the CD8⁺ Tex subset. Through cell communication inference, we propose that *APOE* from Trem tumor cells and *NCF1* from CD8⁺ Tex may constitute a key interaction axis—a proposition further supported at the tissue architecture level by spatial transcriptomic evidence of enhanced Trem–CD8⁺ Tex co-localization and *APOE*–*NCF1* spatial proximity. Finally, by integrating WGCNA, differential expression analysis, and multiple survival modeling strategies, we screened key gene sets associated with prognosis and evaluated the predictive performance of machine learning models across cohorts.

Previous investigations into the immune microenvironment of thyroid carcinoma have predominantly focused on overall immune infiltration levels or a limited set of canonical immune checkpoints. Although single-cell studies are increasingly emerging, research that explicitly couples a “specific tumor cell state” with a “specific Tex subset” and further substantiates this relationship with spatial evidence remains relatively scarce. In contrast, the present study establishes a more cohesive workflow by integrating “cell subset identification — interaction axis proposal — spatial validation — prognostic modeling” into a relatively closed loop. Innovatively, at the tumor cell level, we introduced the novel concept of the Trem subset and contextualized it within an immune-interaction framework. Furthermore, on the immune front, we concentrated on CD8⁺ Tex, elucidating the potential maintenance mechanism of exhaustion through the dual lens of a “putative interaction axis and spatial co-localization.” Ultimately, by bridging these insights into potential mechanisms with prognostic modeling, we explored their potential value for clinical stratification.

However, several aspects of this study warrant careful interpretation. First, there are inherent limitations to in silico cell communication inference: predictions based on transcriptional levels cannot confirm protein-level existence, binding affinity, functional directionality, or causality. The “receptor/ligand” designation also relies on database annotations, which carry inherent uncertainty. Second, spatial resolution constraints: most current spatial transcriptomic technologies capture signals from spots containing multiple cells. While co-localization and distance analyses provide supportive evidence, they do not constitute direct single-cell-level proof of contact. Third, cohort and sample heterogeneity: variations in tissue processing, sequencing depth, and clinical data completeness across different data sources may influence estimates of subpopulation proportions, differential expression analysis, and the generalizability of survival models. Finally, the lack of experimental functional validation: the causal relationship of the *APOE*–*NCF1* axis in suppressing Tex function and promoting tumor progression requires validation through in vitro and in vivo experiments.

In conclusion, this study reveals a potentially critical interactive niche between Trem tumor cells and CD8⁺ Tex in thyroid carcinoma, bolstering its plausibility with spatial evidence, and suggests that the associated molecular network holds prognostic stratification potential. With future validation using higher-resolution spatial technologies and functional experiments, the *APOE*–*NCF1* axis and the Trem–Tex niche may emerge as important breakthroughs for understanding immune evasion and progression mechanisms in thyroid carcinoma and for exploring novel therapeutic strategies.Finally, we suggest that targeting the APOE–NCF1 axis might represent a therapeutic strategy, pending functional studies.

## Supplementary Information

Below is the link to the electronic supplementary material.

Supplementary Material 1

Supplementary Material 2

Supplementary Material 3

Supplementary Material 4

## Data Availability

These data were obtained from the TCGA (https://portal.gdc.cancer.gov/), GEO DataSets (https://www.ncbi.xyz/gds/). We would like to acknowledge them for their free use.
